# Mapping Current and Potential Distribution of Non-Native *Prosopis juliflora* in the Afar Region of Ethiopia

**DOI:** 10.1371/journal.pone.0112854

**Published:** 2014-11-13

**Authors:** Tewodros T. Wakie, Paul H. Evangelista, Catherine S. Jarnevich, Melinda Laituri

**Affiliations:** 1 Graduate Degree Program in Ecology, Colorado State University, Fort Collins, Colorado, United States of America; 2 Natural Resource Ecology Laboratory, Colorado State University, Fort Collins, Colorado, United States of America; 3 US Geological Survey, Fort Collins Science Center, Fort Collins, Colorado, United States of America; 4 Department of Ecosystem Science and Sustainability, Colorado State University, Fort Collins, Colorado, United States of America; University of Vigo, Spain

## Abstract

We used correlative models with species occurrence points, Moderate Resolution Imaging Spectroradiometer (MODIS) vegetation indices, and topo-climatic predictors to map the current distribution and potential habitat of invasive *Prosopis juliflora* in Afar, Ethiopia. Time-series of MODIS Enhanced Vegetation Indices (EVI) and Normalized Difference Vegetation Indices (NDVI) with 250 m^2^ spatial resolution were selected as remote sensing predictors for mapping distributions, while WorldClim bioclimatic products and generated topographic variables from the Shuttle Radar Topography Mission product (SRTM) were used to predict potential infestations. We ran Maxent models using non-correlated variables and the 143 species- occurrence points. Maxent generated probability surfaces were converted into binary maps using the 10-percentile logistic threshold values. Performances of models were evaluated using area under the receiver-operating characteristic (ROC) curve (AUC). Our results indicate that the extent of *P. juliflora* invasion is approximately 3,605 km^2^ in the Afar region (AUC  = 0.94), while the potential habitat for future infestations is 5,024 km^2^ (AUC  = 0.95). Our analyses demonstrate that time-series of MODIS vegetation indices and species occurrence points can be used with Maxent modeling software to map the current distribution of *P. juliflora*, while topo-climatic variables are good predictors of potential habitat in Ethiopia. Our results can quantify current and future infestations, and inform management and policy decisions for containing *P. juliflora*. Our methods can also be replicated for managing invasive species in other East African countries.

## Introduction

Invasive plants are naturalized plants that produce large number of offspring, have the ability for long-distance dispersal, and thus have a potential to spread over a considerable area [1]. Non-native plants, which are synonymous with alien plants and non-indigenous plants, are plant taxa that are introduced to areas beyond their native range through human activity [1], [2]. Invasion by non-native species is among the most critical threats to natural ecosystems worldwide [3]–[6]. *Prosopis* species, commonly known as mesquite, alagarroba, and kiawe, are some of the most highly invasive plants in the world, dominating millions of hectares of arid and semi-arid lands in Africa, Asia, Australia, and the Americas [7], [8]. Historical records show that *Prosopis* was introduced to Sudan in 1917 [9]. There is growing evidence that *Prosopis* species were introduced to Kenya, Somalia, Eritrea, and Ethiopia in the 1970s through collaborative projects involving local governments and international organizations [10], [11]. Today, *Prosopis juliflora*, *P. pallida*, and *P. chilensis* are found in Kenya and Sudan [12], [13]; only *P. juliflora* has been reported in Ethiopia. *Prosopis* hybridizes very rapidly and identification at a species level is often difficult [7], [14]. *Prosopis* species are rapidly spreading in several southern and eastern African countries. In South Africa, for example, hybrid of *Prosopis* is expanding its range at a rate of 18% per annum, doubling its extent every five years [14].

Among the 44 recognized *Prosopis* species, *P*. *glandulosa*, *P. velutina*, *P. juliflora*, and *P. pallida* are the most invasive [7]. In Africa, *Prosopis* species are estimated to have invaded over four million ha, threatening crop and range production, desiccating limited water resources, and displacing native flora and fauna [14], [15]. *Prosopis* species have increased the mortality of *Acacia erioloba*, one of South Africa's important species, by depleting water resources [16]. In Australia, hybrid *Prosopis* species are having dramatic ecological impacts by forming extensive dense stands, and completely excluding native herbs, grasses, and shrubs [17]. Due to its deleterious environmental and economic impacts, the non-native *P. juliflora* has been rated as a very high priority invasive species in Ethiopia [18].

Early detection and mapping of invasive species are essential to formulating effective containment strategies. However, in Ethiopia, quantitative assessments of the area invaded by *P. juliflora* and its potential distribution have not been adequately conducted [19]. Conventional ground surveys and mapping activities are time consuming, and costly, especially over large areas. New integrative spatial modeling approaches that employ advanced remote sensing, Geographic Information Systems (GIS) and modeling algorithms (e.g., correlative models) are increasingly being used to map both the current [20]–[23] and the potential distributions of invasive species [23]. Correlative models include a wide range of machine learning and regression based approaches that attempt to create a relationship between species records (presence/absence), and environmental predictors [24], [25].

Vegetation mapping primarily involves understanding the behavior of the electromagnetic radiation and the reflectance properties of features and plants. Healthy vegetation has chlorophyll which reflects the green, and absorbs the blue and red, portion of the visible electromagnetic radiation. During different phenological stages and stress conditions, the amount of blue and red electromagnetic radiation reflected by plants changes. Likewise, healthy vegetation highly reflects the near-infrared portion of the electromagnetic spectrum. Variation in internal leaf structure among plant species creates subtle differences in reflectance values. This unique spectral value, also called spectral signature, can be detected by remote sensing sensors, and can be used to discriminate plants at a species level [26]. By manipulating reflectance values in the blue, red, and near infrared portion of the spectrum, it is possible to create different ratios and vegetation indices which permit discrimination of vegetated areas. Among the commonly used vegetation indices are the Normalized Difference Vegetation Index (NDVI) [27], [28] and the Enhanced Vegetation Index (EVI) [28], [29]. The NDVI is calculated as:
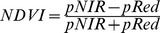
(1)where *pNIR* and *pRed* represent the surface reflectance values of the near-infrared and the red wavelengths, respectively. The EVI is calculated as:
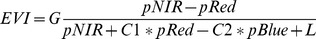
(2)where *pNIR*, *pRed*, and *pBlue* represent the atmospherically or partially atmospherically corrected surface reflectance values of the near-infrared, the red, and the blue wavelengths, respectively. L represents the canopy background factor, while the coefficients C1and C2 are used to correct aerosol scattering in the red band by using the blue band. Generally, Cl = 6, C2 = 7.5, G (gain factor)  = 2.5, and L = 1 [29]. In the United States, both MODIS EVI and NDVI have been used to identify crop lands with high overall accuracy (97%) [30]. The two vegetation indices complement each other in global vegetation studies and improve upon the detection of vegetation changes and extraction of canopy biophysical parameters [29].

*Prosopis juliflora* and *P. pallida* trees have evergreen to semi-evergreen leaves, shedding leaves completely only under stressful and drought conditions [7]. Besides having evergreen leaves, *P. juliflora* forms dense thickets and dominates the canopy layer, all of which are useful traits for remote detection of tree species. Mapping current distributions of invasive plants is generally conducted by discriminating spectral reflectance from different remote sensing sensors and derived vegetation indices [20]–[23]. Recent studies have provided evidence that inclusion of topographic predictors with remote sensing data can improve these mapping efforts (e.g., [31]). In contrast to mapping current distributions, predicting potential distributions attempts to relate species occurrence to environmental conditions, such as climate or topography, and then uses these relationships to predict locations with similar environmental conditions to those where a species is found [32]–[35]. Neither the current nor the potential habitats of invasive *P. juliflora* trees has been quantified in Ethiopia. Here, we present correlative techniques for mapping and modeling both the current and potential distributions of *P. juliflora* trees in Afar (Ethiopia), using remote sensing and topo-climatic predictors, species occurrence points, and Maxent species distribution modeling software [36]. Specifically, our objectives were to: 1) map the current distribution of *P. juliflora* in the Afar region of Ethiopia using a time-series of vegetation indices from Moderate Resolution Imaging Spectroradiometer (MODIS) satellite; and 2) predict its potential distribution using climatic and topographic environmental variables.

## Materials and Methods

### Ethics Statement

Animals were not the subject of this study, and nor were any endangered or protected species. No special permits were required for collecting geographic locations of *P. juliflora* plants from Afar, Ethiopia. The study was approved by appropriate Ethiopian Government Organization – the Afar Pastoral, Agriculture and Rural Development Office (APARDO).

### Study Area

**Figure 1:**
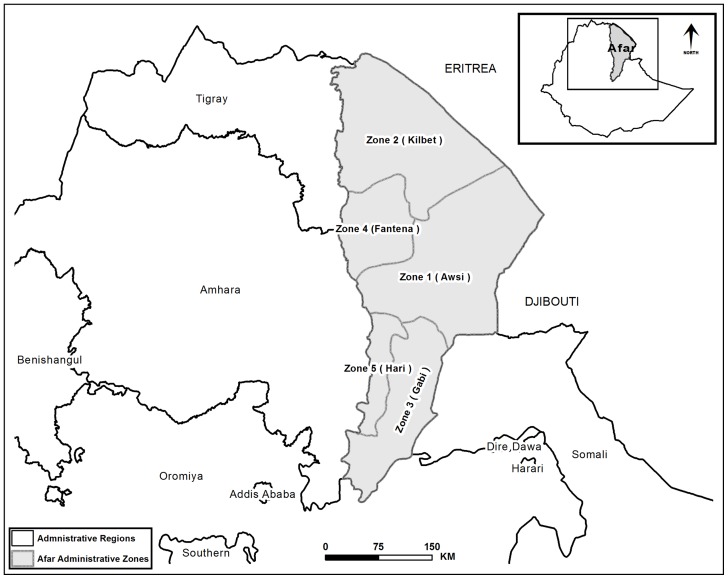
Study Site. Zones are administrative units that are found within *Killils* (regions or states) and can have several *Woredas* (counties). The five zones are referred as Awsi Rasu (Zone 1), Kilbet Rasu (Zone 2), Gabi Rasu (Zone 3), Fantena Rasu (Zone 4) and Hari Rasu (Zone 5).

Our study site is in the Afar Region of the northern part of Ethiopia (between 8° 51′ and 14° 34′ latitudes, and 39° 47′ and 42° 24′ longitudes; Figure 1). The area covers approximately 95,266 km^2^ of land and water, with elevations ranging from 125 m below sea level to 2,870 m above sea level. Long-term climate data (1968–2001) obtained from the Ethiopian Meteorological Agency (EMA) [37] indicates that the mean annual rainfall ranges from 580 mm at Melka Werer to 215 mm at Dufti. The mean maximum annual rainfall recorded for Melka Werer is 673 mm, while the mean minimum annual rainfall recorded at Dufti is 92 mm. The mean annual temperature for Melka Werer and Dufti is 26.6°C and 30.1°C, respectively. The recorded mean minimum annual temperature for Melka Werer is 19.3°C, and mean maximum annual temperature for Dufti is 37.3°C. The study area is located within the *kolla* (arid to semi-arid) and the *bereha* (desert) agro-ecological zones of Ethiopia.

The Afar Region, which is further divided into five smaller administrative zones, is one of the nine administrative regions in Ethiopia. The population living in Afar is estimated at 1,650,000 [38]. Eighty percent of Afar people are pastoralists, while another 10% are considered agro-pastoralist [39]. *Prosopis juliflora* is threatening the livelihoods of Afar pastoralists by displacing native plants that have high livestock grazing and foraging uses. The native vegetation consists of grasses, forbs, shrubs, and woody plants that are adapted to arid and semi-arid environments. The dominant herbaceous (i.e., grasses and forbs) vegetation includes *Chrysopogon*, *Sporobolus*, *Dactyloctenium*, *Cymbopogon*, and *Cynodon* species [40], [41]. The woody vegetation is mainly composed of *Acacia senegal*, *A. nubica*, *A. nilotica*, *A. tortilis*, *A. mellifera*, *Acalypha* species, *Cadaba rotundifolia*, *Dobera glabra*, *Grewia* species, *Salvadora persica*, *Tamarix nilotica*, *Balanites aegyptiaca*, and *Ziziphus spina-christi* [41]–[43]. In addition to livestock, the native plants also provide grazing and foraging uses to the wildlife found in the region. The region contains two national parks (Awash and Yangudi-Rassa), three wildlife reserves (Awash West, Alledeghi, and Mille-Serdo), three controlled hunting areas (Gilen Hertalie, Chifra, and Telalak-Dewe), and one open hunting area (Gelila Dura). The parks and wildlife reserves are homes to the unique wildlife species of Afar including the endangered Grevy's zebra (*Equus grevyi*), and critically endangered wild ass (*E*. *africanus*) [44]–[46].

### Data Collection and Pre-analyses

A total of 143 *P. juliflora* observations with geographic coordinates (presence points) were recorded in 2011 and 2012 in Awsi, Gabi, and Hari Zones. Northern parts of Afar, Kilbet, and Fantena, which border the Tigray and Amhara Region to the west and Eritrea to the north and east, were not sampled due to logistical and security concerns (Figure 1). We followed a targeted sampling approach based on local knowledge. Local communities and government employees, who had detailed knowledge of the local vegetation, landscape, roads, foot-trails, conflict areas, and *P. juliflora* infested sites, facilitated the targeted sampling and data collection process. We covered all known infested sites within the sampled zones. The majority of the occurrence records were 1km apart with a minimum distance of 250 m between occurrence points. In addition to avoiding duplication of sample records, this approach allowed us to reduce spatial autocorrelation.

For the mapping analyses, we selected MODIS products (i.e., MOD13Q1) with 250 m^2^ spatial resolution. Monthly Normalized Difference Vegetation Indices (NDVI) and Enhanced Vegetation Indices (EVI) for the year 2012 were extracted. We obtained all MODIS products from the Land Processes Distributed Active Archive Center (LPDAAC) [47] and conducted all pre-processing (i.e., reprojection, mosaicking and sub-setting) using the MODIS Reprojection Tool (MRT) [48]. For predictive modeling of potential distribution of *P. juliflora*, we used the 19 bioclimatic variables derived from monthly temperature and precipitation values (WorldClim) [49], [50]. The spatial resolution of the bioclimatic predictors for the study site was 0.00833 degrees. Additionally, elevation and slope were obtained from the Shuttle Radar Topography Mission (SRTM) data product [51]. The SRTM products had a spatial resolution of 90 m^2^. All topo-climatic predictors were resampled in ArcGIS 10.0 [52] to 250 m^2^ spatial resolution using the nearest neighborhood algorithm to match the resolution of the remote sensing predictors.

### Data Analyses and Model Evaluation

Maximum entropy modeling software (Maxent; version 3.3.3 k) was selected for mapping the current and potential extent of *P. juliflora* [36]. Maxent is a widely tested correlative model that gives very high predictive accuracy both in terrestrial and marine environments [24]–[25], [53]. Maxent is both a machine learning and statistical method that applies the maximum entropy principle. The maximum entropy principle states that probability distributions should agree with what is known (or inferred from the environmental conditions where the species has been observed), but should avoid assumptions not supported by the data [36], [54]. Maxent thus attempts to find the probability distribution of maximum entropy (i.e., most spread out or close to uniform distribution) subject to constraints imposed by the information available from the observed occurrence records and environmental conditions across the study area [36], [54]–[56]. Unlike other correlative based models that use presence and absence data, Maxent uses presence and background points that assess the available environment for model calibration and testing. We tested all predictors for correlation using presence and background locations in SYSTAT 11.0 software [57]. We removed highly correlated predictors (Pearson correlation coefficient values > +0.8; <−0.8) and variables with low predictive power as measured via percent contribution and variable importance during exploratory analyses.

Two preliminary Maxent models were run; the first with 24 MODIS predictors representing monthly NDVI and EVI, and a second using the 19 available Bioclimatic variables. We identified the best predictor variables based on the percent contribution and permutation importance values provided by Maxent outputs. The preliminary analyses allowed us to reduce the number of variables to eight non-correlated MODIS and six non-correlated Bioclim predictors for mapping distribution and predicting potential habitat, respectively.

For mapping current *Prosopis* distribution, our final variables included NDVI for the months of March, April, September, and November; and EVI for the months of March, October, November, and December. For predicting potential habitat for *Prosopis*, our climate variables were temperature annual range (Bio7), annual precipitation (Bio12), precipitation of wettest month (Bio13), precipitation of driest month (Bio14), precipitation seasonality coefficient of variation (Bio15), and precipitation of coldest quarter (Bio19). In addition, slope and elevation, which also had strong predictive contributions in our preliminary analyses, were included in both of our final models after being subjected to correlative tests (Tables 1 and 2).

**Table 1:** Percent contribution and permutation importance of remote sensing predictors.

Variable name	Percent contribution	Permutation importance
November EVI	43.5	50.0
April NDVI	15.7	10.8
Elevation	12.8	18.7
Slope	6.6	7.3
October EVI	8.2	1.2
March EVI	4.6	1.8
December EVI	2.6	0.8
September NDVI	2.1	1.9
March NDVI	2.0	3.0
November NDVI	1.8	4.4

Maxent model was set to 30% random test percentage and *sub-sample* replication type.

**Table 2:** Percent contribution and permutation importance of topo-climatic predictors.

Variable name	Percent contribution	Permutation importance
Temperature annual range (bio7)	45.9	73.9
Precipitation of wettest month (bio13)	10.1	16.6
Precipitation of coldest quarter (bio19)	12.4	2.0
Slope	9.5	1.7
Precipitation seasonality (bio15)	8.3	2.7
Precipitation of driest month (bio14)	7.5	1.5
Annual precipitation (bio12)	3.8	1.0
Elevation	2.5	0.6

Maxent model was set to 30% random test percentage and *sub-sample* replication type.

The Maxent model allows the user to define or change model parameters beyond the default settings. For our final models, we set the replication type to *sub-sample*, random test percentage to 30%, the number of iterations to 5,000, and the number of replicates to 25. The regularization value in Maxent controls the complexity of the model [36], [58]. We assessed model over-fitting by testing regularization values of 0.5,1, 1.5 and 2. We selected the optimum regularization value of one, which is the default value in Maxent, after visually inspecting response curves for complexity and comparing the train and test AUC (area under the receiver-operating characteristic curve) values.

Sample selection bias is handled in Maxent by manipulating background points during model training and testing. Generating background points in the vicinity of the occurrence records allows both the background and the occurrence points to carry similar types of bias that balance out [55]. Generating background points beyond 100 km distance of occurrence records may result in inflated AUC and simplified predictions [59]. In this study, we randomly generated background points within 50 km distance of the occurrence records. We trained the potential distribution model using the 50 km buffer and made extrapolations (projections) to the entire study site. We selected the *Do clamping* option in Maxent which applies same data ranges for model calibration and extrapolation. Clamping ensures that projection is made using data range found only within the training data set [36], [56]. Predictions into novel environments were assessed using Multivariate Environmental Similarity Surfaces (MESS), which identifies locations which are outside the range of values included in the data used to train the model (the presence and background points) for any predictor [35].

Threshold values used for converting Maxent probability outputs into binary maps can affect the extent of the predicted distribution especially when few number of occurrence records are used and the sampling is biased [60]. Among the four commonly used Maxent threshold values, the 10-percentile training presence produces reliable distributional areas [61]. The 10-percentile threshold miss-classifies 10% of the training presence locations as unsuitable. We converted the probability surfaces generated by the two Maxent models into binary maps using the 10-percentile training presence logistic threshold values and calculated their respective areas. We used large number of occurrence records (143) and we reduced the sampling bias; therefore, the threshold value selected for this study is reasonable.

Model performance was assessed using area under the receiver operating characteristics (ROC) curve (AUC) [62], [63], and maximized Kappa statistic [63], [64]. AUC values ranging from 0.5–0.7, 0.7–0.9, and >0.9 show poor, reasonable, and very good predictions, respectively [62], [65]. Kappa values <0.4, 0.4–0.75, and >0.75 indicate poor, good, and excellent agreements (predictions), respectively [63]. Both AUC and Kappa were calculated using Schröder's ROC-AUC software [66] on independent data sets obtained from the Ethiopian Wildlife Conservation Authority (EWCA; personal communication with Fanuel Kebede). We obtained 50 presence points from EWCA and collected another 50 absence points in December 2013 from the field to validate our results. The test data were evenly distributed across the study site.

## Results

### Current Distribution

**Figure 2:**
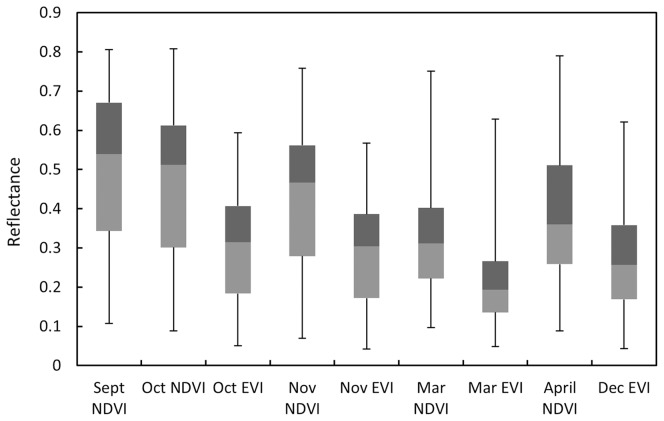
*Prosopis juliflora* reflectance. Box plots of *P. juliflora* EVI and NDVI reflectance values. Note that NDVI and EVI values for the other months were dropped from the final model due to cross-correlations.

**Figure 3:**
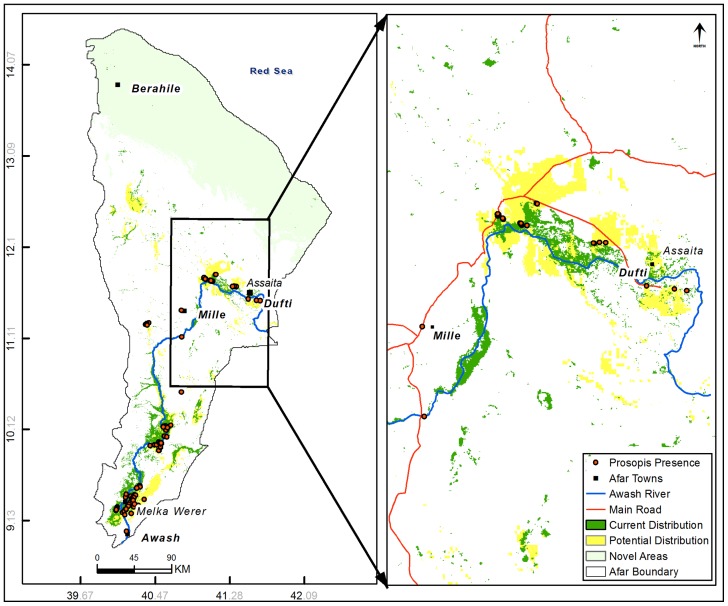
Distribution of *P. juliflora*. The current distribution (shown in green) is superimposed on the potential distribution (shown in yellow). The 143 *P. juliflora* occurrence records used in the model are shown in red. The Multivariate Environmental Similarity Surfaces (MESS) results that indicate areas that are environmentally dissimilar to the training data are shown in light green color.

The remote sensing and topographic predictors with the highest percent contribution for mapping current distributions were November EVI (43.5%), April NDVI (15.7%), elevation (12.8%), and slope (6.6%; Table 1). The NDVI and EVI values for *P. juliflora* showed similar trends with higher values recorded in September, and lower values recorded in March (Figure 2). The NDVI values were always higher than the EVI values. Visual inspection of the current *P. juliflora* distribution map shows that infestation is dominant in the Gabi, Awsi, and Hari administrative zones, respectively (Figure 3). According to the model, the northern most administrative area, Kilbet, is the least invaded. The banks of Awash River are heavily invaded by *P. juliflora* (Figure 3). Area calculations of model results show that the current predicted distribution of *P. juliflora* invasion covers 3,605 km^2^ of the Afar region. The remote sensing and topo-climatic predictors correlated well with the *P. juliflora* occurrence data, with both having high Kappa and AUC values. Kappa and AUC values based on the independent data for the current model were 0.85 and 0.94, respectively (Table 3).

**Table 3:** AUC and Maximized Kappa Statistic values calculated for an independent data set for both the current and the potential distribution models.

Model Type	AUC	Maximized Kappa Statistic
Current Distribution	0.94	0.85
Potential Distribution	0.95	0.86

### Potential Distribution

The topo-climatic predictors with the highest contribution for the potential distribution model were temperature annual range (Bio7; 45.9%), and precipitation of wettest month (Bio13; 10.1%; Table 2). Suitable habitats for *P. juliflora* were predicted throughout the Afar region (Figure 3). The extrapolation assessment (MESS analysis) identified areas of extrapolation (environmental variable values outside the range of those used to train the model) in the northern tip parts of the study site, where the Maxent model did not predict suitable habitats for *P. juliflora* (Figure 3). We are uncertain about the models' prediction in the northern tip of Afar, and thus advise users to interpret our results with caution. Based on area calculations of model results, the potential extent of *P. juliflora* distribution in Afar is 5,024 km^2^. The results show that more than half of the potentially suitable habitats have been invaded. The potential distribution model had an AUC value of 0.95 and a Kappa value of 0.86 based on the independent data set (Table 3).

## Discussion

**Figure 4:**
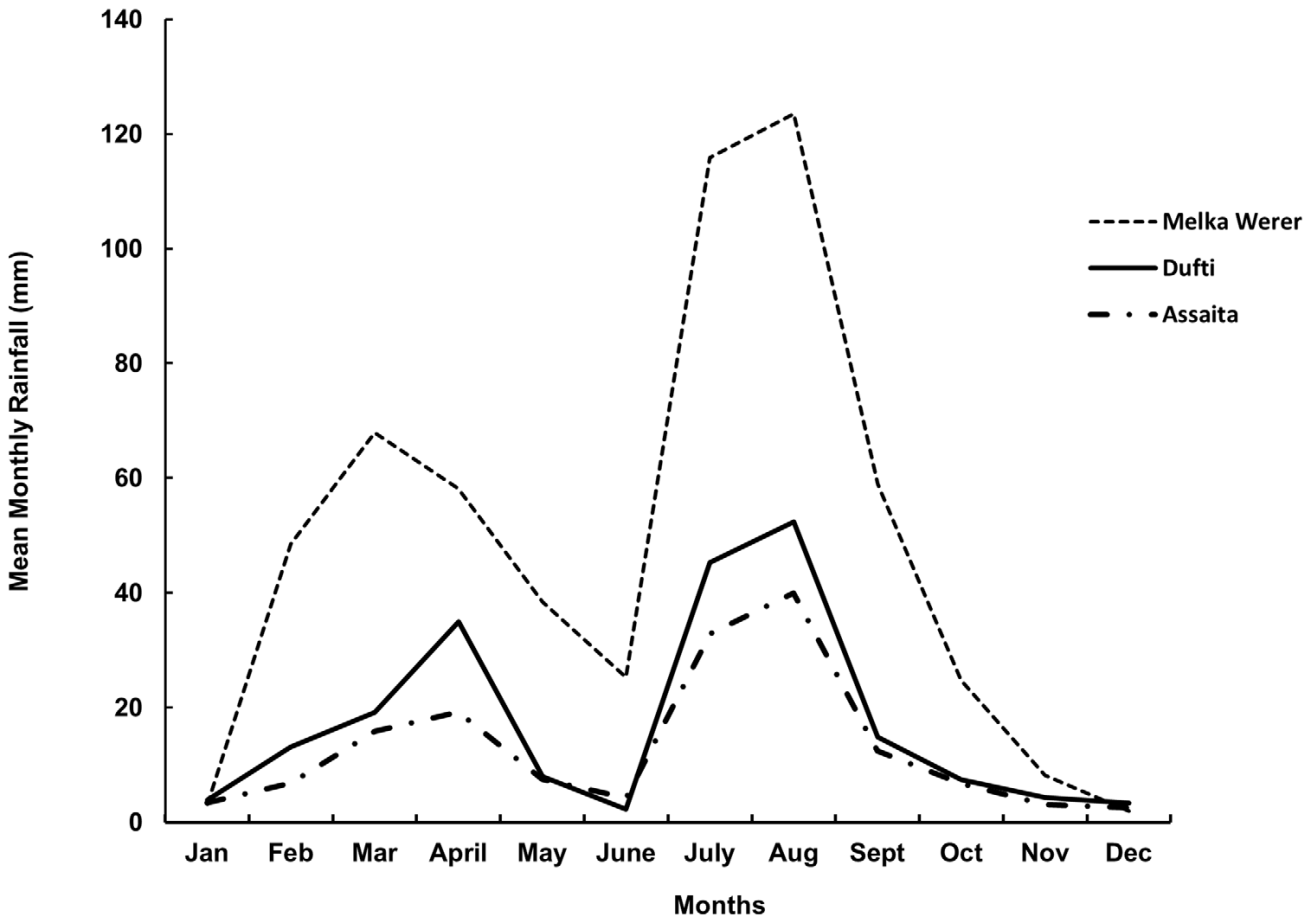
Long term rainfall pattern in Afar. Average mean monthly precipitation for Melka Werer, Dufti, and Assaita stations (1968–2001). The graph shows a distinct S-N aridity gradient between Melka Werer and Assaita.

We found that MODIS Vegetation Indices (VIs) are highly useful for mapping *P. juliflora* in the extensive land of the Afar. The phenological signals of *P. juliflora* were best detected by the November EVI and April NDVI MODIS predictors (Table 1). November represents *hagay* to Afar people, a cold and dry period early in the dry season. During this time, the foliage of most woody shrubs and trees remains green, while herbaceous flora, such as annual grasses and agricultural crops, become less green, creating phenological contrasts for better discrimination of woody vegetation. At the end of the dry season, *P. juliflora* remains green, while woody shrubs and trees lose most of their foliage or take on a yellow coloration due to water stress (personal observation). In addition, *P. juliflora* takes advantage of its deep root systems [67] and the moisture from the short rainy season (between March and April and referred by Afar people as *hugum*) to remain green (Figure 4). These differences were likely detected by the dry season VIs (November, October and December EVIs), and the short rainy season *hugum* VIs (April and March NDVIs, and March EVI). The trend for NDVI and EVI was similar but EVI values were lower (Figure 2). EVI values are generally lower as they avoid saturation in high biomass areas [29]. In mapping current distributions, we hypothesize that EVI was the top predictor because it was able to detect the dense *P. juliflora* thickets that often possess high biomass. Wet season NDVI and dry season EVI predictors highly contributed to the model. The observed seasonal variability among EVI and NDVI predictors in model contribution needs further investigation. Our findings suggest that images taken in November and April are highly useful for remotely detecting *P. juliflora.* In general, our intensive sampling and data collection efforts, the species' distinct canopy architecture and its unique spectral signature have allowed us to detect and map *P. juliflora* trees with acceptable degree of accuracy (Table 3). Our results support the conclusion made by Viña et al. [68] that MODIS vegetation indices can have considerable potential in mapping distributions of species.

Two climate variables appear to best predict the potential distribution of *P. juliflora*. Temperature annual range (Bio7), which is a function of maximum temperature of warmest month and minimum temperature of coldest month, was the most important variable, followed by precipitation of wettest month (Bio13). Our results suggest that temperature and rainfall are important in the distribution of *P. juliflora*. Although slope and elevation did not contribute much in the prediction of potential habitat, they were the third and fourth contributors in mapping current distributions, suggesting incidence of topographic preferences in the distribution of *P. juliflora*. The potential distribution did not cover 100% of the current distribution. This is to be expected when sampling is conducted only in the invaded range, where the invasive species is still expanding and may not be in equilibrium with its environment [54].

The models' high AUC values give us confidence in the overall accuracies of the current and potential distribution maps. However, we believe our model results might be improved if we had the opportunity to sample a wider area within the Afar. We also tested a single correlative modeling approach, Maxent, where other modeling techniques might have produced different results (e.g., Boosted Regression Trees) [69]. Future modeling efforts may consider using samples from the native range for the potential distribution model and using models that can handle both presence and absence data for the current distribution model. Finally, we must recognize the limitations in using coarse-resolution satellite imagery such as MODIS. Detailed modeling using moderate-resolution remote sensing (e.g., Landsat 8, SPOT) and topo-climatic variables may provide more accurate results for smaller geographic areas of interest. For a different perspective on current distribution vs potential distribution (with wildlife examples), and realized-potential niche gradient concept, we advise the reader to refer to Jiménez-Valerde et al. [70], Lobo [71], and Gormley et al. [72].

## Conclusions

We identified suitable habitats for the invasive *P. juliflora* plant throughout the Afar region. Since *P. juliflora* seeds are easily dispersed by domestic and wild animals, streams, and overland water flow [7], [8], [73], we anticipate further expansion of *P. juliflora* invasion into most parts of Afar, Ethiopia. We quantified, for the first time, the current and potential extent of *P. juliflora* invasion in Afar. We found that MODIS vegetation indices and topo-climatic variables can be used with species occurrence data and correlative models to map both the current and potential distribution of *P. juliflora*. The methods described here can be easily applied in other countries that need to monitor invasive species in arid and semi-arid ecosystems. We anticipate that the *P. juliflora* distribution maps that we created will be used as baseline for future monitoring activities, and may inform land managers and policy makers in formulating preventive, control and or eradication measures. Our estimates can also be used to parameterize economic models that may be conducted in the region. Future research should incorporate species presence points from northern parts of Afar and from the species native range. Including soil and hydrologic related predictors in the analyses, using high-resolution time series images and additional species distribution models may also give new insights on the current and potential distribution of *P. juliflora* in Ethiopia.

## References

[1] Richardson DM, Pyšek P, Rejmánek M, Barbour MG, Panetta FD, et al (2000) Naturalization and invasion of alien plants: Concepts and definitions. Diversity and distributions 6: 93–107.

[2] Kolar C, Lodge D (2001) Progress in invasion biology: Predicting invaders. Trends in Ecology & Evolution 16 (4): 199–204. 10.1016/s0169-5347(01)02101-2 11245943

[3] Wilcove D, Rothstein D, Dubow J, Phillips A, Losos E (1998) Quantifying threats to imperiled species in the United States. American Institute of Biological Sciences 48: 607–615.

[4] Sharma G, Singh J, Raghubanshi A (2005) Plant invasions: Emerging trends and future implications. Current Science 88: 726–734.

[5] Butchart S, Walpol M, Collen B, van Strien A, Scharlemann J, et al (2010) Global biodiversity: Indicators of Recent Declines. Science 328: 1164–1168. 20430971 10.1126/science.1187512

[6] Simberloff D, Martin JL, Genovesi P, Maris V, Wardle D, et al (2013) Impacts of biological invasions: What's what and the way forward. Trends in Ecology & Evolution 28 (1): 58–66. 10.1016/j.tree.2012.07.013 22889499

[7] Pasiecznik N, Felker P, Harris P, Harsh L, Cruz G, et al. (2001) The *Prosopis juliflora* – *Prosopis pallida* complex: A monograph. HDRA, Coventry, UK. 172 p.

[8] Gallahar T, Merlin M (2010) Biology and impacts of Pacific Island invasive species. 6. *Prosopis pallida* and *Prosopis juliflora* (Algarroba, Mesquite, Kiawe) (Fabaceae). Pacific Science 64: 489–526.

[9] Brown A, Massey R (1929) Flora of the Sudan. Thomas Murby and CO. 376 p.

[10] Zollner D (1986) Sand dune stabilization in central Somalia. Forest Ecology and Management 16: 223–232.

[11] Coppock D, Aboud A, Kisoyan P (2005) Agro-pastoralists' wrath for the *Prosopis* tree: The case of the Il Chamus of Baringo district, Kenya. Research Brief 05-02-PARIMA. Global Livestock Collaborative Research Support Program, University of California Davis 3 p.

[12] Choge S, Pasiecznik N, Harvey M, Wright J, Awan S, et al (2007) *Prosopis* pods as human food with special reference to Kenya. Water SA 33: 419–424.

[13] Salah O, Yagi S (2011) Nutritional composition of *Prosopis chilensis* (Molina) Stuntz leaves and pods from Sudan. African Journal of Food Science and Technology 2: 79–82.

[14] Zimmermann H, Hofmann J, Witt A (2006) A South African Perspective on *Prosopis* . Biocontrol News and Information 27: 6–9.

[15] Witt A (2010) Biofuels and invasive species from an African perspective – a review. GCB Bioenergy 2: 321–329.

[16] Schachtschneider K, February E (2013) Impact of *Prosopis* invasion on a keystone tree species in the Kalahari Desert. Plant Ecology 214: 597–605.

[17] van Klinken R, Graham J, Flack L (2006) Population ecology of hybrid mesquite (*Prosopis* species) in western Australia: How does it differ from native range invasions and what are the implications for impacts and management? Biological Invasion 8: 727–741.

[18] Ethiopian Flora Network (2010) Invasive plants. Available: http://www.etflora.net/databases/invasive-plants. Accessed 30 Sep 2013.

[19] Mauremootoo J (2006) Current status and future prospects for *Prosopis juliflora* in Ethiopia. Biocontrol news and information 27: 37–40.

[20] Evangelista P, Stohlgren T, Morisette J, Kumar S (2009) Mapping invasive Tamarisk (Tamarix): A comparison of single-scene and time-series analysis of remotely sensed data. Remote Sensing 1: 519–533.

[21] Zimmermann H, Wehrden H, Damascos M, Bran D, Welk E, et al (2011) Habitat invasion risk assessment based on Landsat 5 data, exemplified by the shrub *Rosa rubiginosa* in southern Argentina. Austral Ecology 36: 870–880.

[22] Li W, Guo Q (2010) A maximum entropy approach to one-class classification of remote sensing imagery. International Journal of Remote Sensing 31 (8): 2227–2235.

[23] Bradley B, Olsson A, Wang O, Dickson B, Pelech L, et al (2012) Species detection vs. habitat suitability: Are we biasing habitat suitability models with remotely sensed data? Ecological Modeling 244: 57–64.

[24] Elith J, Graham H (2009) Do they? How do they? Why do they differ? On finding reasons for differing performances of species distribution models. Ecography 32: 66–77.

[25] Elith J, Graham C, Anderson R, Dudik M, Ferrier S, et al (2006) Novel methods improve prediction of species' distribution from occurrence data. Ecography 29: 129–151.

[26] Lillesand T, Kiefer R, Chipman J (2008) Remote sensing and image interpretation. United States: WILEY Press. 756 p.

[27] Tucker C (1979) Red and photographic infrared linear combinations for monitoring vegetation. Remote Sensing of Environment 8: 127–150.

[28] Huete A, Liu H, Batchily, Leeuwen W (1997) A comparison of vegetation indices over a global set of TM images for EOS-MODIS. Remote Sensing of Environment 59: 440–451.

[29] Huete A, Didan K, Miura T, Rodriguez E, Gao X, et al (2002) Overview of the radiometric and biophysical performance of MODIS vegetation indices. Remote Sensing of Environment 83: 195–213.

[30] Wardlow B, Egbert S (2013) A comparison of MODIS-250m EVI and NDVI data for crop mapping: A case study for Southwest Kansas. International Journal of Remote Sensing 31 (3): 805–830.

[31] von Wehrden H, Zimmermann H, Hanspach J, Ronnenberg K, Wesche K (2009) Predictive mapping of plant species and communities using GIS and Landsat data in a southern Mongolian mountain range. Folia Geobot 44: 211–225.

[32] Ficetola G, Thuiller W, Miaud C (2007) Prediction and validation of the potential global distribution of a problematic alien invasive species-the American bullfrog. Diversity and Distributions 13: 476–485.

[33] Ward D (2007) Modelling the potential geographic distribution of invasive ant species in New Zealand. Biol Invasions 9: 723–735.

[34] Evangelista P, Kumar S, Stohlgren T, Jarnevich C, Crall A, et al (2008) Modelling invasion for a habitat generalist and specialist species. Diversity and Distributions 14: 808–817.

[35] Elith J, Kearney M, Phillips S (2010) The art of modelling range-shifting species. *Methods in Ecology and Evolution* 1: 330–342.

[36] Phillips S, Anderson R, Schapire R (2006) Maximum entropy modeling of species geographic distributions. Ecological Modeling 190: 231–259.

[37] Ethiopian Metrological Agency (EMA) (2012) Available: http://www.ethiomet.gov.et. Accessed 2013 Jun 16.

[38] Central Statistical Agency (CSA) (2012) Available: http://www.csa.gov.et. Accessed 2013 May 20.

[39] Environmental Protection Authority of Ethiopia (EPA) (2010) Afar national regional state program of plan and adaptation to climate change. Available: http://www.epa.gov.et. Accessed 2012 May 25.

[40] Abule E, Snyman H, Smit G (2007) Rangeland evaluation in the middle Awash valley of Ethiopia: I. Herbaceous vegetation cover. Journal of Arid Environments 70: 253–271.

[41] Pastoralist Forum Ethiopia (PFE) (2010) Pastoralism and land: Land tenure, administration and use in pastoral areas of Ethiopia. Available: http://www.pfe-ethiopia.org/pub.html. Accessed 2013 Sep 30.

[42] Abule E, Snyman H, Smit G (2007) Rangeland evaluation in the middle Awash valley of Ethiopia: II. Woody vegetation. Journal of Arid Environments 70: 253–271.

[43] Tikssa M, Bekele T, Kelbessa E (2009) Plant community distribution and variation along the Awash River corridor in the main Ethiopian rift. African Journal of Ecology 48: 21–28.

[44] Kebede F, Bekele A, Moehlman P, Evangelista P (2012) Endangered Grevy's zebra in the Alledeghi Wildlife Reserve, Ethiopia: Species distribution modeling for the determination of optimum habitat. Endangered Species Research 17: 237–244.

[45] Ethiopian Wildlife Conservation Authority (2012) Available: http://www.ewca.gov.et. Accessed 2014 Jul 26.

[46] World Wildlife Fund (2014) Available: http://www.worldwilldlife.org/ecoregions/at1305. Accessed 2014 Jul 30.

[47] USGS Earth Resources Observation and Science Center, Sioux Falls, South Dakota. Available: https://lpdacc.usgs.gov/data_acess. Accessed 2013 Feb 12.

[48] MODIS Reprojection Tool (MRT) (2011) Available: https://lpdaac.usgs.gov/tools/modis_reprojection_tool. Accessed 2012 Jan 20.

[49] Hijmans R, Cameron S, Parra J, Jones P, Jarvis A (2005) Very high resolution interpolated climate surface for global land areas. International Journal of Climatology 25: 1965–1978.

[50] BioClim, Version 1.4. Available: http://www.worldclim.org. Accessed 2013 Jan 11.

[51] Shuttle Radar Topography Mission (SRTM) (2010) available: http://srtm.usgs.gov/. Accessed 2012 May 10.

[52] ESRI (2012) Desktop Release 10. Redlands, CA: Environmental Systems Research Institute.

[53] Reiss H, Cunze S, Konig K, Neumann H, Kroncke I (2011) Species distribution modelling of marine benthos: A North Sea case study. Marine Ecology Process Series 442: 71–86.

[54] Peterson A, Soberon J, Pearson R, Anderson R, Martinez-Meyer E, et al. (2011) Ecological niche and geographic distribution. Princeton and Oxford: Princeton University Press. 314 p.

[55] Phillips S, Dudik M (2008) Modeling of species distribution with Maxent: New extensions and a comprehensive evaluation. Ecography 31: 161–175.

[56] Elith J, Phillips S, Hastie T, Dudik M, Chee Y, et al (2011) A statistical explanation of MaxEnt for ecologists. Diversity and Distributions 17: 43–57.

[57] SYSTAT (2008) Version 11. San Jose, CA, USA.

[58] Anderson R, Gonzalez I (2011) Species-specific tuning increases robustness to sampling bias in models of species distributions: An implementation with Maxent. Ecological Modelling 222: 2796–2811.

[59] VanDerWal J, Shoo L, Graham C, Williams S (2009) Selecting pseudo-absence data for presence only distribution modeling: How far should you stray from what you know. Ecological modeling 220: 589–594.

[60] Bean W, Stafford R, Brashares J (2012) The effect of small sample size and sample bias on threshold selection and accuracy assessment of species distribution models. Ecography 35: 250–258.

[61] Escalante T, Rodriguez-Tapia G, Linaje M, Illolidi-Rangel P, Gonzalez-Lopez R (2013) Identification of areas of endemism from species distribution models: Threshold selection and Nearctic mammals. Tip Revista Especializada en Ciencias Quimico-Biologicas 16 (1): 5–17.

[62] Hanley J, McNeil B (1982) The meaning and use of Area under a Receiver Operating Characteristic (ROC) Curve. Radiology 143: 29–36. 7063747 10.1148/radiology.143.1.7063747

[63] Fielding A, Bell J (1997) A review of methods for assessment of prediction errors in conservation presence/absence models. Environmental Conservation 24 (1): 38–49.

[64] Cohen J (1960) A coefficient of agreement for nominal scales. Educational and Psychological Measurements XX (1): 37–46.

[65] Swets J (1988) Measuring the accuracy of diagnostic systems. Science 240: 1285–1293. 3287615 10.1126/science.3287615

[66] Schröder B (2006) ROC plotting and AUC calculation transferability test. Available: http://brandenburg.geoecology.uni-potsdam.de/users/schroeder/download.html. Accessed 2013 Mar 8.

[67] Canadell J, Jackson R, Ehleringer J, Mooney H, Sala O, et al (1996) Maximum rooting depth of vegetation types at the global scale. Oecologia 108: 583–595. 28307789 10.1007/BF00329030

[68] Viña A, Bearer S, Zhang H, Ouyang Z, Liu J (2006) Evaluating MODIS data for mapping wildlife habitat distribution. Remote Sensing of Environment 112: 2160–2169.

[69] Elith J, Leathwick R, Hastie T (2008) A working guide to boosted regression trees. Journal of Animal Ecology 77: 802–813. 18397250 10.1111/j.1365-2656.2008.01390.x

[70] Jiménez-Valerde A, Lobo JM, Hortal J (2008) Not as good as they seem: The importance of concepts in species distribution modelling. Diversity and Distributions 14: 885–890.

[71] Lobo J (2008) More complex distribution models or more representative data? Biodiversity Informatics 5: 14–19.

[72] Gormley A, Forsyth D, Griffioen P, Lindeman M, Ramsey D, et al. (2011) Using presence-only and presence-absence data to estimate the current and potential distributions of established invasive species. 10.1111/j.1365-2664.2010.01911.x PMC3038347 21339812

[73] Shiferaw H, Teketay D, Nemomissa S, Assefa F (2004) Some biological characteristics that foster the invasion of *Prosopis juliflora* (Sw.) DC. at middle Awash rift valley area, north-eastern Ethiopia. Journal of Arid Environments 58: 135–154.

